# Regulation of Mitotic Exit by Cell Cycle Checkpoints: Lessons From *Saccharomyces cerevisiae*

**DOI:** 10.3390/genes11020195

**Published:** 2020-02-12

**Authors:** Laura Matellán, Fernando Monje-Casas

**Affiliations:** Centro Andaluz de Biología Molecular y Medicina Regenerativa (CABIMER), Spanish National Research Council (CSIC)—University of Seville—University Pablo de Olavide, Avda, Américo Vespucio, 24, 41092 Sevilla, Spain; lauramatellanfer@gmail.com

**Keywords:** mitosis, checkpoint, DNA damage, chromosome segregation, aneuploidy

## Abstract

In order to preserve genome integrity and their ploidy, cells must ensure that the duplicated genome has been faithfully replicated and evenly distributed before they complete their division by mitosis. To this end, cells have developed highly elaborated checkpoints that halt mitotic progression when problems in DNA integrity or chromosome segregation arise, providing them with time to fix these issues before advancing further into the cell cycle. Remarkably, exit from mitosis constitutes a key cell cycle transition that is targeted by the main mitotic checkpoints, despite these surveillance mechanisms being activated by specific intracellular signals and acting at different stages of cell division. Focusing primarily on research carried out using *Saccharomyces cerevisiae* as a model organism, the aim of this review is to provide a general overview of the molecular mechanisms by which the major cell cycle checkpoints control mitotic exit and to highlight the importance of the proper regulation of this process for the maintenance of genome stability during the distribution of the duplicated chromosomes between the dividing cells.

## 1. Introduction

**Figure 1:**
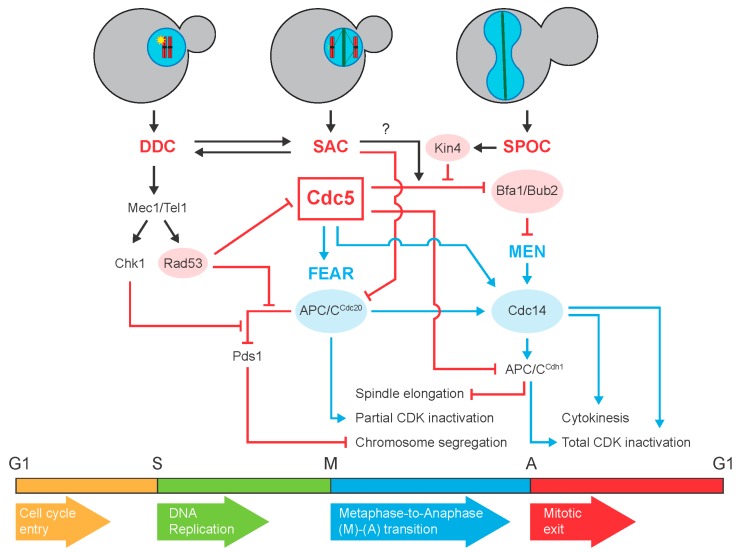
Diagram summarizing the main signaling pathways by which the DNA damage (DDC), the spindle assembly (SAC) and the spindle position (SPOC) checkpoints restrain cell cycle progression after their activation due to DNA lesions (depicted as a yellow star), unattached chromosomes (marked with yellow lines emanating from the unbound kinetochore) or an incorrect spindle alignment, respectively. The scheme also outlines the interplay between these surveillance mechanisms and the polo-like kinase Cdc5 to ensure the integrity and the even distribution of the duplicated genome during mitosis. Interactions are indicated by lines that end in an arrow when positive or in a bar when negative. Blue lines and red lines highlight, respectively, positive and negative signaling events that are important for the regulation of mitotic exit by the cell cycle checkpoints. FEAR: Cdc-fourteen early anaphase release; MEN: mitotic exit network.

Exit from mitosis is the final cell cycle transition, which represents the completion of mitosis and the entry into a new interphase. This process requires a tight coordination of multiple signaling pathways to ensure a faithful distribution of the genomic material and the cellular content between the dividing cells. Problems during mitotic exit or defects due to its deregulation can lead to changes in the chromosome number of a cell that can give rise to aneuploidy (an important hallmark of cancer), genetic diseases and neurodegenerative disorders [1,2,3,4,5]. Exit from mitosis is determined by the inactivation of the complexes that cyclin-dependent kinases (CDKs) establish with their associated cyclin subunits, which promote the initiation and the transition through the different stages of the cell cycle [6,7]. Cyclin/CDK inactivation is carried out by the anaphase-promoting complex/cyclosome (APC/C), which induces cyclin degradation by the proteasome in association with two cofactors: Cdc20, which triggers a first wave of CDK inactivation in metaphase, and Cdh1, which acts later in mitosis [6,8,9]. An additional key event for mitotic exit in budding yeast is the release of the protein phosphatase Cdc14 from the nucleolus, which takes place in a stepwise manner: first into the nucleus and then throughout the cell. Once released, Cdc14 determines the de-phosphorylation of mitotic CDK substrates, which eventually leads to their inactivation and the accumulation of the CDK-inhibitor Sic1, thereby triggering mitotic exit and cytokinesis [10,11,12,13]. The liberation of Cdc14 from the nucleolus is regulated by two different signaling cascades: the FEAR (Cdc-fourteen early anaphase release), which promotes early release of the phosphatase from the nucleolus to the nucleus at the metaphase-to-anaphase transition, and the MEN (mitotic exit network), which finally triggers the complete release of Cdc14 to the cytoplasm [10,14,15,16] (Figure 1). While the FEAR release of Cdc14 is dispensable for budding yeast cell viability, the MEN-dependent full liberation of the phosphatase is required for completion of mitosis. The MEN regulates both mitotic exit and cytokinesis in *Saccharomyces cerevisiae,* and it is closely related to the Hippo pathway in animal cells [10,15,16,17]. The most upstream component of the MEN is the Tem1 GTPase, which activates a signaling cascade that includes the Cdc15 and Dbf2-Mob1 kinases and mediates Cdc14 final release. The activity of Tem1 is negatively regulated by the two-component GTPase-activating complex (GAP) Bfa1-Bub2 and positively controlled by the Lte1 protein [18,19].

The execution of mitotic exit is tightly and precisely coordinated and controlled to prevent cell division being completed before chromosomes have been correctly replicated and equally distributed between the mother and the daughter cell. As such, different surveillance mechanisms or checkpoints that delay or inhibit cell cycle progression to ensure the faithful inheritance of the genomic material specifically impinge on this cell cycle transition. In *S. cerevisiae*, the main mitotic checkpoints are the DNA damage checkpoint (DDC), which is triggered by lesions to the genomic material, the spindle assembly checkpoint (SAC), which responds to unattached kinetochores, and the spindle position checkpoint (SPOC), which prevents exit from mitosis until the spindle is properly positioned. Remarkably, and although the previous checkpoints are triggered by different insults or intracellular signals and are activated at different cell cycle stages, all of them actively promote and depend on the inhibition of mitotic exit signaling (Figure 1). Interestingly, a key cell cycle regulator that mediates this general checkpoint-dependent inhibition of mitotic exit is the Cdc5 protein, the only polo-like kinase (Plk) homolog to mammalian Plk1 in budding yeast [20,21,22]. Cdc5 has a pivotal role both in the regulation of the FEAR and the MEN signaling pathways and also for the functionality of the main mitotic checkpoints [23,24,25,26] (Figure 1). In this review, we provide an updated overview of the control of mitotic exit by the main cell cycle checkpoints and discuss the role of Cdc5 as a central regulator that facilitates the coordination among them to ensure a successful completion of mitosis.

## 2. The DNA Damage Checkpoint

The maintenance of genome integrity is essential to preserve the viability of the cells. Several external factors such as ionizing or ultraviolet radiation and chemical agents can modify the DNA structure. Additionally, lesions to the DNA can originate from endogenous factors, including the generation of reactive oxygen species (ROS) as a consequence of their own cellular metabolism, or due to the processes of chromosome replication, transcription, repair or segregation. In the presence of DNA damage, cells activate a checkpoint response that is globally known as the DNA damage response (DDR) [27,28]. Within the DDR, a major mechanism that coordinates DNA repair with cell cycle progression is the evolutionary conserved DDC, which delays or restrains cell cycle progression in response to DNA damage so that cells have enough time as to repair the lesions to the genomic material [29,30,31]. In most eukaryotic cells, DDC activation impairs mitotic entry. However, in *S. cerevisiae,* this checkpoint specifically impinges on the metaphase-to-anaphase transition while, additionally, also promoting mitotic exit inhibition [32,33,34,35]. It is worth noting that the DDC differs from the DNA replication checkpoint (DRC), another surveillance mechanism that is specific to S phase and is activated in response to arrested replication forks [30,36,37]. Although both checkpoints act in concerted action as part of a general intra-S checkpoint [38], this is beyond the focus of this review.

The main sensors of the DDC are the protein kinases Mec1 and Tel1 (ATR and ATM in mammals, respectively), which in response to DNA lesions, directly phosphorylate the adaptor proteins Rad9 and Mrc1, thereby activating the DDC-effector kinases Chk1 and Rad53 (Chk1 and Chk2 in animal cells, respectively) [36,39,40,41,42,43,44,45,46]. In *S. cerevisiae*, Chk1 and Rad53 participate in two separate and parallel branches of the DDC to restrain cell cycle progression after DNA damage [32,33,47]. Accordingly, lack of both checkpoint effectors has an additive impact in the DDC-dependent cell cycle arrest. In budding yeast, activation of Chk1 and Rad53 blocks the cell cycle at the metaphase-to-anaphase transition [32,47]. In the presence of DNA damage, Chk1 phosphorylates Pds1 (securin), thereby preventing its ubiquitination by the APC/C-Cdc20 complex and its subsequent degradation by the proteasome [48,49,50]. Since Pds1 acts as an inhibitor of Esp1 (separase), a protease that cleaves the cohesin complexes that keep sister chromatids together, its DDC-dependent Chk1 activation inhibits sister chromatid separation, this way promoting the metaphase arrest [33,48]. Pds1 stabilization and Esp1 inhibition further contribute to this arrest by blocking FEAR activation, as separase is necessary to support the function of this signaling pathway and the early release of Cdc14 [51,52]. Rad53 also promotes maintenance of Pds1 stability, and hence, the metaphase block by preventing the Pds1 and Cdc20 interaction [50], likely through the direct phosphorylation of Pds1 by Rad53 after DDC activation [53] (Figure 1).

Interestingly, the activation of Rad53 in response to DNA damage also halts elongation of the mitotic spindle [54]. During an unperturbed cell cycle, the concerted action of the Cdc5 and Cdk1 kinases controls spindle elongation through phosphorylation and inhibition of the Cdh1 cofactor of the APC/C. Inactivation of APC/C-Cdh1 allows accumulation of the microtubule motor proteins Cin8 and Kip1, therefore promoting the elongation of the mitotic spindle [55]. Remarkably, however, the activation of the DDC triggers a Rad53-dependent phosphorylation of Cdc5 that inhibits the polo-like kinase, thus favoring Cdh1 activity and subsequently also restraining spindle elongation and anaphase progression [34,54] (Figure 1). Accordingly, overexpression of Cdc5 enables mitotic spindle elongation after DDC activation, significantly shortening the cell cycle arrest [32,54]. These evidences highlight the relevance of the polo-like kinase Cdc5 in the regulation of the DDC.

As previously emphasized, despite Chk1 and Rad53 activation leading to a cell cycle block at the metaphase-to-anaphase transition, the DDC also simultaneously promotes the inhibition of mitotic exit [32,33,34,35]. Specifically, stimulation of Rad53 kinase activity inhibits MEN signaling by acting on the Bfa1-Bub2 complex. Under normal growth conditions, Cdc5 phosphorylates and inhibits the GAP component Bfa1 during anaphase, triggering the activation of MEN signaling and promoting mitotic exit [35,56]. However, the generation of DNA lesions at the telomeres and the subsequent activation of the DDC in *cdc13-1* mutant cells determines a Rad53-dependent inhibition of the polo-like kinase that leads to the inactivation of Tem1 and mitotic exit [34] (Figure 1). These mutants accumulate single-stranded DNA at the restrictive temperature due to problems in telomere “capping”. Puzzlingly, and although MEN inhibition is observed after exposure of cells to different types of DNA damage, it seems to only be required for cell viability after telomeres are damaged but not in response to other chromosomal lesions, suggesting a specific role of this particular branch of the DDC in the protection of the cells when telomere integrity is compromised [34].

Fascinatingly, a recent study has revealed that the DDC can be activated in response to DNA double-strand breaks (DSBs) even in telophase, thus reinforcing the idea that a central regulatory target of this checkpoint is the mitotic exit process [57]. As such, generation of DSBs during telophase triggers an activation of the DDC that determines a delay in the transition from telophase to G1 characterized by a partial reversion of sister chromatid segregation and the coalescence of sister chromatid loci. Interestingly, this process seems to depend on the regulation of the activity of the Cin8 kinesin motor protein by the DDC, which promotes its dephosphorylation and redistribution to spindle pole bodies (SPBs) and/or kinetochores [57].

After the transient cell cycle arrest in response to DNA damage, the DDC is inactivated through a recovery process once that the lesion is finally repaired. However, inactivation of the DDC can also occur through an adaptation process when the damage persists over time and cells cannot resolve the DNA lesion [58]. The bypass of the DDC-dependent cell cycle arrest by adaptation, originally described in *S. cerevisiae*, likely represents a way to allow the survival of at least a certain fraction of the cells in a population, even at the expense of promoting genome instability, in contrast to a stable arrest that would lead to a loss of the whole population [59,60,61]. Remarkably, both DDC recovery and adaptation rely on Rad53 deactivation. The molecular mechanisms guiding DDC recovery are still far from being completely understood, although several factors besides the Rad53 kinase have been identified, including the H2A or H4 histones and different PP2A protein phosphatase complexes, among others [62,63,64,65,66,67]. Interestingly, Cdc5 seems not to play any role in the recovery from DDC activation in budding yeast [68,69]. However, during DNA damage adaptation, this kinase displays a pivotal function mediating the inactivation of Rad53 [59,70,71,72,73]. Specifically, it has been shown that Cdc28/Cdk1 promotes high levels of Cdc5 activity during DNA damage adaptation, thereby determining the inhibition of Cdh1 and Bfa1 by the polo-like kinase and enabling the elongation of the mitotic spindle, progression through the cell cycle and, eventually, mitotic exit even in the presence of persistent DNA lesions [74,75,76]. Despite the central role of Cdc5 in the adaptation to DNA damage, the precise molecular mechanisms by which the polo-like kinase controls this process still require further investigation.

Finally, it is important to mention that, besides the DDC, there are additional mechanisms that regulate mitotic exit after DNA damage. In this sense, it has been described the existence of an interplay between the protein kinase A (PKA) pathway and the DDC in order to prevent the onset of anaphase in response to DNA lesions in budding yeast [77,78]. PKA is a tetrameric complex whose activation depends on the presence of cyclic-AMP (cAMP) [79]. Remarkably, in response to DNA damage, Mec1 and cAMP trigger an activation of PKA that promotes the phosphorylation of Cdc20 and the subsequent inhibition of the APC/C. Additionally, this pathway further promotes the stabilization of high levels of Pds1, which, as a whole, restrains anaphase onset and exit from mitosis [77,78,80,81].

## 3. The Spindle Assembly Checkpoint

The accurate and faithful segregation of the genomic material between the dividing cells requires the correct attachment of all chromosomes to the spindle, a bipolar array of microtubules that allows for their distribution during mitosis. The microtubules from the spindle specifically bind to the kinetochore, a protein complex assembled on the centromeres of the chromosomes [82]. In *S. cerevisiae*, each kinetochore is stably attached to the plus-end of only one microtubule in metaphase, unlike multiple microtubules simultaneously binding to the same kinetochore in humans [83]. Binding of all kinetochores to spindle microtubules is, however, not the only condition for the equitable partition of the genomic content during mitosis. Its even distribution further requires the bi-orientation of the chromosomes, which implies binding of both sister chromatids (i.e., the two copies of a duplicated DNA molecule) of each chromosome to microtubules emanating from opposite poles of the mitotic spindle [83]. To ensure that both requirements are fulfilled, most eukaryotic cells have developed two fundamental surveillance mechanisms. As such, and in the first instance, cells trigger the spindle assembly checkpoint (SAC) [84,85,86], an evolutionary conserved system that detects failures in chromosomal attachment and blocks the cell cycle in metaphase until all kinetochores are bound to microtubules emanating from the spindle (reviewed in [87,88]). Additionally, cells count with an error correction mechanism that senses, destabilizes and corrects kinetochore-microtubule attachments that do not lead to the bi-orientation of chromosomes and in which the protein kinase Aurora B, together with the other members from the chromosomal passenger complex, plays a fundamental role (reviewed in [89,90]). Defects in the functionality of these surveillance mechanisms can end up with an incorrect distribution of the genomic material during mitosis, and hence, lead to aneuploidy and initiate tumorigenic processes [91,92]. Indeed, several strategies are currently being developed that target SAC activity as anticancer and tumor suppression treatments [93,94,95].

An empty kinetochore as a consequence of a failure in its attachment to the spindle microtubules promotes the recruitment of SAC proteins to this structure and the subsequent activation of the checkpoint. This generates a “wait-anaphase” signal that inhibits the activity of the APC/C-Cdc20 complex and thus impedes the separation of sister chromatids and blocks the metaphase-to-anaphase transition (Figure 1). Remarkably, the presence of even a single unattached kinetochore is sufficient to activate the SAC and efficiently restrain cell cycle progression [96,97]. The SAC targets and sequesters the Cdc20 cofactor, and hence, negatively regulates the APC/C-dependent ubiquitination and destruction of cyclin B and securin by the proteasome [98,99,100]. Specifically, activation of the SAC promotes the formation of a Cdc20 inhibitory complex known as the MCC (mitotic checkpoint complex), which is a hetero-tetramer formed by the conserved proteins Mad2, Bub3 and Mad3/BUBR1, as well as by Cdc20 itself [101,102]. Remarkably, conformational changes in Mad2 during SAC activation that both increase the affinity of this protein for Cdc20 and allow it to form protein complexes that further activate additional inactive cytoplasmic Mad2 molecules lead to an amplification of the “wait-anaphase” SAC signal through a positive feedback loop, which generates a strong cell cycle arrest through sequestration of Cdc20 and inhibition of APC/C activity [87,103,104]. Furthermore, the incorporation of Cdc20 into the MCC promotes its own ubiquitination by the APC/C in order to degrade the excess of Cdc20 that is not inhibited by the SAC, and thus, to more strongly maintain the mitotic arrest [105].

As indicated previously, in addition to the SAC, Aurora B kinases also have an essential role in safeguarding the fidelity of chromosome segregation by ensuring the bi-orientation of the chromosomes acting in coordination with the SAC in the error correction pathway [89,106]. Many of the aspects of the mechanisms by which Aurora B kinases contribute to the repair of incorrect kinetochore-microtubule attachments have been elucidated in budding yeast. The bi-orientation of the chromosomes generates tension on the sister kinetochores as a consequence of the microtubules from opposite spindle poles pulling from both chromatids of the same chromosome. Ipl1, the Aurora B homolog in *S. cerevisiae*, is able to detect the lack of tension in the mitotic spindle due to incorrect chromosomal attachments and to subsequently phosphorylate substrates at the kinetochores that promote a destabilization of the erroneous microtubule attachments, thus generating empty kinetochores that activate the SAC [107,108,109,110]. Remarkably, Ipl1 is so efficient in correcting erroneous chromosomal attachments during the early stages of mitosis that the SAC, which is essential for cell viability in most higher eukaryotes, is only required in budding yeast when the Aurora B homolog activity is somehow compromised [111]. Besides Aurora B, the centromeric protein Shugosin (Sgo1) is also important to sense the lack of tension as a consequence of incorrect kinetochore-microtubule attachments and to promote the bi-orientation of the chromosomes [112,113]. The mechanisms by which erroneous chromosomal attachments are resolved are beyond the scope of this review and are extensively summarized elsewhere [89,114].

In the same way as the DDC, the proper function of the SAC also requires the inhibition of MEN signaling and the active prevention of Cdc14 nucleolar release. Consequently, the absence of Bfa1 or Bub2, similarly to that of Mad2 or other SAC proteins, causes the cell to override the metaphase block in response to unattached kinetochores [115,116,117]. However, lack of Bfa1-Bub2 does not interfere with the SAC-dependent inhibition of APC/C-Cdc20 [117]. After activation of the SAC, Bfa1 remains hypo-phosphorylated and active in a partially Cdc5-dependent manner, and this contributes to inhibition of the MEN, and thus, of mitotic exit [35,118]. However, in the case of the SAC, the molecular mechanisms leading to this inhibition still remain unclear (Figure 1).

Remarkably, although the SAC induces an inhibition of Bfa1 phosphorylation by Cdc5 in budding yeast, polo-like kinases play an important role in the functionality of this checkpoint. As such, in human cells, Plk1 phosphorylates and inhibits Cdc20 at the kinetochores in cooperation with the Bub1 kinase, which acts as a scaffold for the polo kinase at this location and with Aurora B. This leads to the inactivation of the APC/C complex in an MCC-independent manner and, hence, to the maintenance of SAC signaling and the checkpoint-dependent metaphase arrest [119,120]. Plk1 bound to Bub1 further contributes to the stabilization of kinetochore-microtubule attachments and the activity of the SAC [121]. Plk1 also acts together with Mps1, a conserved SAC kinase essential for MCC assembly and SAC signal amplification, to strengthen and maintain checkpoint signaling [122]. Moreover, besides the prior functions, polo kinases are required for the recruitment of many components of the SAC and for correct checkpoint signaling [88]. Specifically, Cdc5, in collaboration with Ipl1, is needed for Mad3/BubR1 phosphorylation during SAC activation in *S. cerevisiae* [123]. Therefore, kinases from the polo family play a fundamental role in the functionality of the SAC that seems to be highly conserved in most eukaryotes by regulating the activity and/or localization of essential SAC components at the kinetochores and by inhibiting the APC/C-Cdc20 complex.

Interestingly, it has been recently proposed that the SAC contributes to the response to DNA damage in order to maintain genome integrity (Figure 1). When DDC signaling is compromised by the lack of Mec1 and Tel1, the SAC becomes essential to prevent the erroneous segregation of incompletely replicated or damaged chromosomes [124]. The SAC further shows an interplay with the DDC to contribute to restrain cell cycle progression after DDC activation through the inhibition of the APC/C-Cdc20 complex (Figure 1). In this context, the Mad2 protein plays a pivotal role but, unlike during a normal SAC response, a functional kinetochore is not necessary, and it is instead a centromere-dependent mechanism that mediates Mad2 function in this process [125,126]. Additionally, the SAC can trigger Rad53 activation in a Mps1 and Bub1-dependent manner [127]. These evidences highlight an interesting observation regarding these surveillance mechanisms: besides inhibiting the stage of mitosis particularly targeted by each specific checkpoint, they further restrain subsequent phases of the mitotic process by activating other checkpoints with the capacity to block these cell cycle transitions in the case that the initial checkpoint is eventually overridden before the problem can be solved. This agrees with the main surveillance mechanisms that maintain genome stability and its faithful distribution specifically targeting exit from mitosis, which represents the last cell cycle stage that can be prevented to provide the cells with the time to fix errors that could affect the integrity or segregation of the chromosomes.

After all the chromosomes are correctly and stably attached, the SAC must be rapidly silenced to allow entry into anaphase and mitotic exit. In *S. cerevisiae*, deactivation of this checkpoint depends on the PP1 phosphatase, which is recruited to kinetochores based on its interaction with the integral component of this structure, Spc105. Once at the kinetochores, PP1 counteracts the phosphorylation of Bub1-Bub3, Mps1 and Ipl1 substrates in order to stabilize the correct bi-oriented chromosomal attachments [128,129,130,131,132]. SAC-silencing at the kinetochores eventually involves the dissipation of the “wait-anaphase” signal, which is mediated by the release of Cdc20 from the MCC and the subsequent activation of the APC/C, thus allowing the cell cycle to progress into anaphase.

## 4. The Spindle Position Checkpoint

During the asymmetric division of *S. cerevisiae*, cell polarity and the cleavage plane are defined and predetermined before the assembly of the mitotic spindle. Hence, it is essential to correctly position and align the spindle parallel to the mother-daughter cell polarity axis and perpendicular to the cytokinesis plane in order to ensure a proper distribution of the chromosomes during mitosis. As a consequence, budding yeast cells have developed the SPOC, a surveillance mechanism that delays cell cycle progression when the spindle is not correctly positioned by inhibiting MEN signaling, and therefore, preventing mitotic exit until one SPB enters the bud and the segregation of half of the duplicated genome into the daughter cell is ensured [133,134]. The main effector of the SPOC is the Kin4 kinase, which regulates both the activity and the localization of the Bfa1-Bub2 GAP complex on the SPBs in response to spindle misalignment [133,134] (Figure 1). The control of Kin4 distribution in the cell is essential for proper SPOC function. Interestingly, Kin4 asymmetrically localizes exclusively to the mother cell cortex and to the bud neck, while it is excluded from the daughter cell. Additionally, it also transiently localizes to the SPB retained in the mother cell during anaphase, while it is excluded from the daughter cell SPB by the MEN activator Lte1 [133,134,135,136,137]. The integral SPB component Spc72 acts as a scaffold for both Kin4 and Bfa1 on these structures [138,139].

In cells with mispositioned spindles, Kin4 loads on both SPBs, which are retained within the mother cell cytoplasm [134,137,140]. Similarly, the Bfa1-Bub2 complex also changes from its asymmetric distribution on the daughter-destined SPB to be symmetrically localized to both SPBs under these circumstances [18,141,142]. Once on both SPBs, Kin4 phosphorylates Bfa1, thus preventing its inhibitory phosphorylation by Cdc5 and maintaining the MEN in an inactive state [134,137,138]. When the spindle is misaligned, Bfa1 is further phosphorylated by mitotic CDK, which acts in parallel to Kin4 in order to maintain an active GAP complex and prevent exit from mitosis until proper spindle reposition [143]. Bfa1 phosphorylation by Kin4 increases the dynamics of exclusion of the Bfa1-Bub2 complex from the SPBs [137,142]. This enhanced dynamicity is further promoted by association of phosphorylated Bfa1 to the 14-3-3 protein Bmh1 [144]. The simultaneous action of Kin4 and Bmh1 impairs Bfa1 association to Spc72, therefore preventing its phosphorylation by Cdc5 [139]. Remarkably, the increased dynamicity of Bfa1-Bub2 at the SPBs also determines the exclusion from the SPBs of the MEN effector Tem1, whose localization to these structures depends on the GAP complex and is essential for MEN signaling, further contributing to the inhibition of mitotic exit [145]. Importantly, the dynamicity of Bfa1-Bub2 on the SPBs is essential for SPOC proficiency, as demonstrated by constitutive tethering of the GAP to the SPBs impairing the SPOC response [146].

Due to their importance for the proper function of the SPOC and the maintenance of cell ploidy under conditions that obstruct the positioning of the spindle, the activity and localization of Kin4 during the cell cycle are tightly regulated. In this way, the bud neck-associated Elm1 kinase phosphorylates the Kin4 activation loop to promote its activity, while the ubiquitin-ligases Dma1 and Dma2 promote Elm1 recruitment to the bud neck to facilitate its function in SPOC activation [147,148,149]. On the other hand, localization of Kin4 to both the mother cell cortex and SPBs is controlled by the PP2A-Rts1 phosphatase, which maintains the kinase in an hypo-phosphorylated state [140]. Thus, Elm1 and Rts1 act as positive regulators of the SPOC. Conversely, Lte1 is a key regulator of Kin4 localization that directly interacts and excludes the SPOC kinase from the bud, hence acting as an inhibitor of the checkpoint [135,136]. Lte1 exclusively localizes to the daughter cell cortex, and its overexpression or abnormal localization in the mother cell abrogates the SPOC-mediated cell cycle arrest [18,19,135,136,150]. Lte1 binds and inactivates Kin4 in vitro, although this inhibition has not been demonstrated in vivo using *lte1*Δ mutant cells [135]. Remarkably, Lte1 has also been suggested to contribute to counteracting PP2A function [135].

Based on the antagonistic role and distribution of Kin4 and Lte1 during cell division, a “zonal model” has been proposed to explain how spindle positioning and mitotic exit are temporally and spatially coordinated [151]. According to this model, the mother cell compartment acts as a MEN inhibitory zone where Kin4 maintains this signaling cascade in an inactive state. Conversely, the daughter cell represents an activating zone in which Lte1 inhibits Kin4 and allows MEN activation and exit from mitosis once one SPB enters the compartment. Supporting this model, the analysis of genetically-modified cells with two mitotic spindles, one misaligned and the other correctly positioned with respect to the bud neck, allowed to demonstrate that entry of one SPB into the daughter cell determines a signal that triggers mitotic exit and the disassembly of both spindles, despite one of them being incorrectly aligned [139,152]. In addition to this MEN-inhibitory signal, mitotic CDK activity must be kept at low levels to promote MEN activation and facilitate mitotic exit [153]. Interestingly, it has been recently shown that the FEAR pathway triggers MEN activation in the mother cell compartment in cells with misaligned spindles by promoting the phosphorylation of Bfa1 and Cdc15 and that the key function of Kin4 in the SPOC is to counterbalance this FEAR-dependent activation of the MEN [143]. Accordingly, Kin4 function seems to become dispensable for the functionality of the SPOC in cells that also lack the FEAR [143].

Currently, the precise molecular mechanism by which the misalignment of the mitotic spindle is sensed and this signal is transmitted to Kin4 in order to activate the SPOC remains unclear. Among other factors, astral microtubules could play a pivotal role in this process. The lack of interaction between astral microtubules and the bud cortex has been proposed to promote SPOC activation [142]. However, the SPBs seem to also play a fundamental function in this signaling system. In this way, since Kin4 directly interacts with Spc72, which is the cytoplasmic receptor for the γ-tubulin complex at the SPBs [138], the SPOC kinase could additionally be somehow directly receiving information about the orientation of the mitotic spindle at this location.

## 5. Interplay between the Mitotic Checkpoints: A Central Role for Polo Kinases

During cell division by mitosis, there is a tight coordination between progression through the different cell cycle stages with the permanent scrutiny of the faithful duplication, integrity and even distribution of the chromosomes, so that in case genome stability or ploidy is compromised, the mitotic checkpoints can restrain cell cycle progression until the problem is fixed. As previously stated, although the DDC, the SAC and the SPOC are induced in response to different stimuli and at different phases of the cell cycle, there is a crosstalk between all these surveillance mechanisms. Such an interplay is especially evident at mitotic exit, a process that is under the regulation of all the previous checkpoints. Remarkably, Plks turned out to be a central hub to coordinate the regulation of mitotic exit by the main cell cycle checkpoints.

Plks constitute a highly evolutionary conserved protein family with fundamental functions as cell cycle regulators [154,155]. Paradoxically, these kinases have the capacity to both promote and inhibit cell cycle progression. A paradigmatic example of the pleiotropic roles played by these kinases in cell cycle control is Cdc5, the only Plk in budding yeast, which among many other functions favors entry into mitosis by acting as a negative regulator of the Swe1 kinase and by promoting the function of the Clb2 cyclin [156,157]. Cdc5 also becomes crucial during the metaphase-to-anaphase transition for a correct chromosome segregation, since it promotes cleavage of the cohesin ring [158], as well as to regulate the dynamics of spindle elongation [159,160]. As previously stated, and importantly for the functionality of the cell cycle checkpoints, Cdc5 additionally plays an essential role in the regulation of mitotic exit by acting at different levels to control the proper execution of this final cell cycle transition. In this sense, Cdc5 contributes to Cdh1 activation, and thus, to APC/C functionality during the last stages of mitosis. Furthermore, the polo-like kinase triggers activation of both the FEAR and MEN pathways during anaphase, promoting Cdc14 release and activation [10]. Finally, Cdc5 kinase activity is also important to cytokinesis [161,162,163,164].

The multiple functions of Cdc5 in cell cycle control, as well as its fundamental role in the proficiency and interconnection between the different mitotic checkpoints, are timely and spatially coordinated by changes in the subcellular localization of the polo-like kinase throughout the cell cycle, which can be differentially found in the nucleus, the yeast centrosomes and in the bud neck at different cell cycle stages [161,165,166,167]. Additionally, Cdc5 is also regulated through phosphorylation by different kinases, such as Cdc28/Cdk1 or PKA, which act as general upstream regulators of the Plk protein family [155]. Remarkably, Cdk1-dependent phosphorylation of the T242 residue of budding yeast Cdc5 is crucial for its kinase activity, while that of the T70 site is important for MEN activation [75,168]. Interestingly, although phosphorylation of T238 in the T-loop of Cdc5 is not essential for cell viability in *S. cerevisiae*, mutations in this residue lead to defects in its kinase activity, in chromosome stability and in the process of DDC adaptation [74,75]. In agreement with the evolutionary conservation shown by proteins of the Plk family, the functions of these kinases in higher eukaryotes are regulated by similar mechanisms. As such, modification of the phosphorylation status of several residues in Plk1 homologs from different higher eukaryotes have been shown to modulate their role in the control of APC/C activity, their recruitment to kinetochores in response to Aurora B and SAC activation or their function in DDC recovery after Aurora A-mediated phosphorylation [169,170,171,172].

In *S. cerevisiae*, as previously mentioned, an important point of convergence by which the cell cycle checkpoints regulate mitotic exit is the inhibition of the Cdc5-dependent phosphorylation of the Bfa1-Bub2 complex, which leads to the inactivation of this GAP, in order to restrain MEN signaling, and thus, prevent mitotic exit (Figure 1). Each checkpoint, however, promotes this inhibition through a different mechanism. In this sense, it is well established that the inhibitory phosphorylation of Bfa1 by Cdc5 is prevented after SPOC activation through the concerted action of Kin4 and Bmh1 [137,138,139], while in response to DNA damage, the inhibition of Bfa1 phosphorylation is promoted by the DDC kinase Rad53, which inactivates Cdc5 kinase activity to block mitotic exit signaling [34]. Remarkably, during the mitotic arrest induced after DNA damage, Cdc5 and Bfa1 localize at distinct sides of the SPBs (to the nuclear and the cytoplasmic side, respectively) [167]. Therefore, Cdc5 remains spatially separated from Bfa1-Bub2, which also contributes to prevent mitotic exit after DDC activation. Finally, SAC activation could also perturb Cdc5 activity and distribution to prevent Bfa1 phosphorylation. As such, defects in kinetochore-microtubule attachments promote an inhibition of the Cdc5-dependent phosphorylation of Bfa1 mediated by Mad2 and Mps1, and, accordingly, Cdc5 overexpression overrides the cell cycle arrest induced after SAC activation [35]. However, in this case, the molecular mechanisms are still not completely understood.

All the above, in summary, demonstrate that cells have developed an exquisitely elaborate and complex signaling system to ensure the maintenance of genome stability and a correct ploidy during their division in which the different surveillance mechanisms controlling the integrity, faithful duplication and even distribution of the chromosomes are temporally and spatially coordinated and tightly interconnected. In response to problems during these processes, the mitotic checkpoints restrain cell cycle progression to provide the cells with time to repair the damage. Remarkably, and although each surveillance mechanism fundamentally promotes cell cycle arrest at a specific mitotic stage, they further inhibit additional cell cycle transitions, exit from mitosis being a fundamental target of all the main cellular checkpoints. Finally, the evidences shown in this review also emphasize the pivotal role of the kinases from the Plk family in the functionality and the interplay between the different mitotic checkpoints and, hence, in the maintenance of genome stability during cell division. Accordingly, Plks are highly overexpressed in several types of cancer, and these kinases are considered as promising targets for anticancer therapies [173,174,175,176]. It is worth noting, in this sense, that based on the elevated degree of evolutionary conservation, the analysis of Cdc5 functions and substrates in budding yeast have been and will still be in the future of great importance to extend our knowledge about the mechanisms by which Plks regulate cell cycle progression and checkpoint activity in higher eukaryotes, and thus, to potentially develop future new cures for cancer.
